# *SSD1* modifies phenotypes of Elongator mutants

**DOI:** 10.1007/s00294-019-01048-9

**Published:** 2019-11-27

**Authors:** Fu Xu, Anders S. Byström, Marcus J. O. Johansson

**Affiliations:** 1 Västerbotten County Council/Department of Odontology, Umeå University, 901 87 Umeå, Sweden; 2 Department of Molecular Biology, Umeå University, 901 87 Umeå, Sweden

**Keywords:** Elongator complex, tRNA modification, Translation, mRNA-binding protein

## Abstract

The translational decoding properties of tRNAs are influenced by post-transcriptional modification of nucleosides in their anticodon region. The Elongator complex promotes the first step in the formation of 5-methoxycarbonylmethyl (mcm^5^), 5-methoxycarbonylhydroxymethyl (mchm^5^), and 5-carbamoylmethyl (ncm^5^) groups on wobble uridine residues in eukaryotic cytosolic tRNAs. Elongator mutants in yeast, worms, plants, mice, and humans not only show a tRNA modification defect, but also a diverse range of additional phenotypes. Even though the phenotypes are almost certainly caused by the reduced functionality of the hypomodified tRNAs in translation, the basis for specific phenotypes is not well understood. Here, we discuss the recent finding that the phenotypes of *Saccharomyces cerevisiae* Elongator mutants are modulated by the genetic background. This background-effect is largely due to the allelic variation at the *SSD1* locus, which encodes an mRNA-binding protein involved in post-transcriptional regulation of gene expression. A nonsense *ssd1* allele is found in several wild-type laboratory strains and the presence of this allele aggravates the stress-induced phenotypes of Elongator mutants. Moreover, other phenotypes, such as the histone acetylation and telomeric gene silencing defects, are dependent on the mutant *ssd1* allele. Thus, *SSD1* is a genetic modifier of the phenotypes of Elongator-deficient yeast cells.

## Introduction

Post-transcriptionally modified nucleosides are found within all tRNA molecules. Modified nucleosides in the anticodon region usually promote proper anticodon-codon interactions and they are consequently important for the efficiency and fidelity of translation (Agris et al. 2017; Björk and Hagervall 2014). Uridine residues present at the wobble position (nucleoside 34) in eukaryotic cytosolic tRNAs are frequently modified to an xm^5^U-type of modified nucleoside where the xm^5^ moiety is either a 5-methoxycarbonylmethyl (mcm^5^), 5-methoxycarbonylhydroxymethyl (mchm^5^), or 5-carbamoylmethyl (ncm^5^) group (Machnicka et al. 2014). The xm^5^U residues sometimes also contain an additional 2′-*O*-methyl (xm^5^Um) or 2-thio (xm^5^s^2^U) group. The presence of an xm^5^U_34_, xm^5^Um_34_, or xm^5^s^2^U_34_ residue is generally believed to improve pairing with the cognate codon(s) (Agris et al. 2017; Björk and Hagervall 2014; Björk et al. 2007; Johansson et al. 2008; Lim 1994). In this review, we discuss the phenotypic consequences of the lack of wobble xm^5^ groups in *Saccharomyces cerevisiae*, focusing on the recent finding that the phenotypes are modulated by the genetic background.

**Fig. 1:**
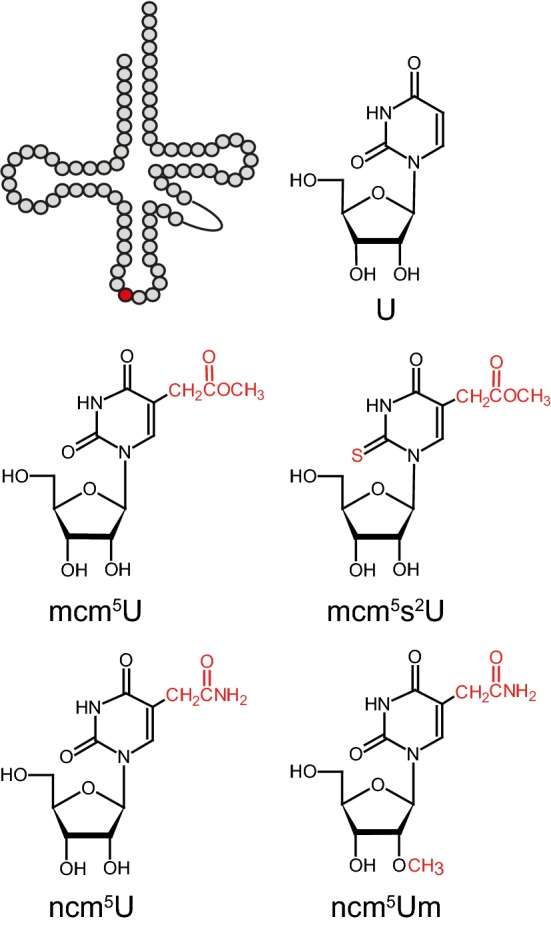
Schematic secondary tRNA structure and the chemical structures of U, mcm^5^U, mcm^5^s^2^U, ncm^5^U, and ncm^5^Um. The wobble position and posttranscriptional modifications are indicated in red

The first step in formation of the xm^5^ groups is dependent on the Elongator complex, which is composed of two sets of the six Elp proteins (Elp1–Elp6) (Dauden et al. 2017, 2018; Huang et al. 2005; Johansson et al. 2018; Kolaj-Robin and Seraphin 2017; Setiaputra et al. 2017; Winkler et al. 2001). Elongator is thought to catalyze the formation of a cm^5^U_34_ residue, which is then further modified by additional enzymes. The xm^5^ moiety found in cytosolic *S. cerevisiae* tRNAs is either an mcm^5^ or ncm^5^ group. Such groups are present in 11 U_34_-containing tRNA species of which two carry mcm^5^U_34_, three mcm^5^s^2^U_34_, five ncm^5^U_34_, and one ncm^5^Um_34_ (Fig. 1) (Johansson et al. 2008 and references therein). In addition to the lack of mcm^5^/ncm^5^ groups in the 11 tRNAs (Huang et al. 2005; Johansson et al. 2008), the inactivation of yeast Elongator leads to a wide range of phenotypes. These phenotypes include slow growth as well as increased sensitivity to various stress-inducing substances and conditions (Frohloff et al. 2001; Karlsborn et al. 2014; Otero et al. 1999). Moreover, Elongator mutants have been reported to show defects in histone acetylation, RNA polymerase II transcription, telomeric gene silencing, mitochondrial function, exocytosis, and protein homeostasis (Li et al. 2009b; Nedialkova and Leidel 2015; Otero et al. 1999; Rahl et al. 2005; Tigano et al. 2015; Winkler et al. 2002). All of these phenotypes, except for the tRNA modification defect, are suppressed by increased expression of various combinations of ${\text{tRNA}}_{\text{UUU}}^{\text{Lys}}$, ${\text{tRNA}}_{\text{UUG}}^{\text{Gln}} ,$ and ${\text{tRNA}}_{\text{UUC}}^{\text{Glu}}$ which are the three *S. cerevisiae* tRNA species that normally carry a mcm^5^s^2^U_34_ residue (Chen et al. 2011; Esberg et al. 2006; Nedialkova and Leidel 2015; Tigano et al. 2015). These findings indicate the lack of the mcm^5^/ncm^5^ groups preferentially affects the functionality of ${\text{tRNA}}_{\text{UUU}}^{\text{Lys}}$, ${\text{tRNA}}_{\text{UUG}}^{\text{Gln}} ,$ and ${\text{tRNA}}_{\text{UUC}}^{\text{Glu}}$ and that the phenotypes of Elongator mutants are caused by inefficient decoding of the respective cognate codons. This notion is further supported by the observation that the inactivation of the Ncs2/Ncs6 complex, which catalyzes the formation of the s^2^ group, induces essentially the same phenotypes that are also suppressed by increased expression of the ${\text{tRNA}}_{\text{UUU}}^{\text{Lys}}$, ${\text{tRNA}}_{\text{UUG}}^{\text{Gln}} ,$ and ${\text{tRNA}}_{\text{UUC}}^{\text{Glu}}$ combinations (Björk et al. 2007; Chen et al. 2011; Esberg et al. 2006; Huang et al. 2008; Leidel et al. 2009; Nakai et al. 2008; Noma et al. 2009). Moreover, ribosome profiling experiments have shown that the lack of wobble mcm^5^/ncm^5^ and/or s^2^ groups leads to an accumulation of ribosomes with AAA, CAA, and GAA codons in their A-site (Chou et al. 2017; Nedialkova and Leidel 2015; Zinshteyn and Gilbert 2013). The mechanism by which the inefficient decoding of these codons induces the phenotypes are not well understood. One model suggests that the phenotypes may be caused by reduced expression of factors encoded from mRNAs enriched in AAA, CAA, and/or GAA codons (Bauer et al. 2012; Chen et al. 2011; Fernandez-Vazquez et al. 2013; Rezgui et al. 2013). In this model, the slower decoding of the mRNA leads to reduced protein abundance by a mechanism that may involve elevated levels of frameshifting or inhibition of translation initiation through ribosome queuing. Another model suggests that the phenotypes may be caused by the proteotoxic stress that arises from defects in co-translational protein folding and the consequent accumulation of protein aggregates (Nedialkova and Leidel 2015). As the proteins that show increased aggregation in strains lacking the mcm^5^/ncm^5^ and s^2^ groups are not encoded by mRNAs enriched in AAA, CAA, and/or GAA codons (Nedialkova and Leidel 2015), it remains unclear if the protein aggregation is a direct or indirect consequence of the inefficient decoding of these codons.

The recent finding that the phenotypes of Elongator-deficient cells are influenced by the allelic variant at the *SSD1* locus provides additional information into the pleiotropic effects of Elongator (Xu et al. 2019). Several wild-type laboratory *S. cerevisiae* strains harbor a nonsense mutation in the *SSD1* gene, which encodes an mRNA-binding protein that associates with a subset of mRNAs and regulates their stability, translation, and/or localization (Hogan et al. 2008; Jansen et al. 2009; Jorgensen et al. 2002; Kurischko et al. 2011; Ohyama et al. 2010; Sutton et al. 1991; Uesono et al. 1997; Wanless et al. 2014). The notion that the *SSD1* locus influences the phenotypes of Elongator mutants was inferred from the observation that the temperature sensitivity (Ts) of cells deleted for *ELP3*, which encodes an Elongator subunit, is significantly stronger in the W303 than in the related S288C genetic background (Xu et al. 2019). Strains in the W303 genetic background contain the nonsense *ssd1*-*d2* allele whereas those in S288C harbor an *SSD1* allele that encodes the full-length functional protein (Jorgensen et al. 2002; Sutton et al. 1991). Analyses of congenic *ssd1*-*d2 elp3Δ* and *SSD1 elp3Δ* strains, in both genetic backgrounds, showed that the *ssd1*-*d2* allele not only aggravates the Ts phenotype of *elp3Δ* mutants but also the growth defects induced by various stress-inducing agents (Xu et al. 2019). In these assays, the effect of the *ssd1*-*d2* mutation is comparable to an *ssd1Δ* allele. Further, the telomeric gene silencing and histone H3 acetylation defects of W303-derived Elongator mutants were found to be dependent on the *ssd1*-*d2* allele, i.e., the phenotypes are suppressed by the introduction of the *SSD1* gene.

The *SSD1* gene has been genetically implicated in a diverse range of cellular pathways and processes, including cell morphogenesis, cell wall integrity, cellular aging, virulence, several signal transduction pathways, protein homeostasis, and transcription by RNA polymerase I, II, and III (Jorgensen et al. 2002; Kaeberlein et al. 2004; Kaeberlein and Guarente 2002; Stettler et al. 1993; Wheeler et al. 2003; Wilson et al. 1991). A likely explanation to the large number of genetic interactions is the function of Ssd1 in post-transcriptional gene regulation. For the Ssd1-associated mRNAs that encode factors involved in cell wall biosynthesis, Ssd1 is thought, depending on its phosphorylation status, to promote either translational repression or polarized localization (Jansen et al. 2009; Kurischko et al. 2011; Wanless et al. 2014). Moreover, the inactivation of *SSD1* alters the abundance and stability of many mRNAs and this effect is not restricted to Ssd1-associated transcripts (Jansen et al. 2009; Li et al. 2009a). The precise mechanisms by which the allele at the *SSD1* locus influences the phenotypes of Elongator mutants are not known, but they may involve both direct and indirect effects of Ssd1´s function in messenger ribonucleoprotein complexes. The *ssd1*-*d2* allele does not influence the formation of the mcm^5^/ncm^5^ groups and analyses of the *ssd1*-*d2 elp3Δ* and *SSD1 elp3Δ* strains revealed no apparent difference in tRNA levels or the abundance of other modified nucleosides (Xu et al. 2019). Further, +1 frameshifting assays indicated that the A-site selection rate at the AAA codon is comparable in *ssd1*-*d2 elp3Δ* and *SSD1 elp3Δ* strains. While these observations suggest that the *ssd1*-*d2* allele does not influence the abundance or functionality of the hypomodified tRNAs, it remains possible that the lack of Ssd1 may affect tRNA function under stress conditions. However, the two phenotypes that are dependent on the *ssd1*-*d2* allele, the histone H3 acetylation and telomeric gene silencing defect, are detected under standard growth conditions, indicating that at least these phenotypes are not caused by a synergistic effect on tRNA function. The telomeric gene silencing defect in Elongator mutants is thought to be caused by reduced levels of the Sir4 protein, which is involved in the assembly of silent chromatin (Chen et al. 2011). The *SIR4* open reading frame is enriched in AAA codons and the telomeric gene silencing defect is suppressed by increased expression of ${\text{tRNA}}_{\text{UUU}}^{\text{Lys}}$ (Chen et al. 2011). Further, the overexpression of ${\text{tRNA}}_{\text{UUU}}^{\text{Lys}}$ restores Sir4 levels without significantly influencing the mRNA levels (Chen et al. 2011). Even though these observations imply that the decreased silencing at telomeres is caused by reduced Sir4 levels, it is not known if the reduction is large enough to cause the phenotype and if it is a direct consequence of inefficient decoding of the *SIR4* mRNA. Nevertheless, the finding that the telomeric gene silencing defect is dependent on the *ssd1*-*d2* allele shows that the lack of the mcm^5^/ncm^5^ groups is not sufficient to induce the phenotype (Xu et al. 2019). Additional experiments are needed to investigate if the levels of Sir4 are modulated by the allele at the *SSD1* locus.

The effect of the *SSD1* locus also partially explained why an *elp3Δ ncs2Δ* double mutant, which lacks both the mcm^5^/ncm^5^ and s^2^ groups, is nonviable in the W303 but not in the S288C genetic background (Björk et al. 2007; Klassen et al. 2015; Nedialkova and Leidel 2015; Xu et al. 2019). An *ssd1*-*d2elp3Δ ncs2Δ* strain is, however, viable but very slow-growing in the S288C background, indicating the growth phenotype is influenced by additional genetic factors (Xu et al. 2019). Consistent with the finding that Ssd1 promotes Hsp104-mediated protein disaggregation (Mir et al. 2009), the levels of aggregated proteins were found to be higher in *ssd1*-*d2elp3Δ ncs2Δ* than in *SSD1elp3Δ ncs2Δ* cells (Xu et al. 2019). Although these observations indicate that the slow growth of the *ssd1*-*d2elp3Δ ncs2Δ* strain may be caused by the accumulation of protein aggregates, it is not known if the aggregation is the cause or the consequence of the growth defect.

The presence of the nonsense *ssd1*-*d2* allele sensitizes yeast cells to the translational defects induced by the lack of Elongator-dependent tRNA modifications. Future work is needed to define the mechanisms by which *SSD1* modulates the phenotypes of Elongator-deficient cells. It also remains to be determined if the phenotypes of Elongator mutants in other organisms are modulated by polymorphisms in genes for mRNA-binding proteins.
